# Study of the Formation of Neuromodulatory Compounds in Fermented Products Based on Triticale, Naked Oat, and Amaranth Grains

**DOI:** 10.3390/foods15173146

**Published:** 2026-09-04

**Authors:** Svetlana Kamanova, Gaukhar Akshorayeva, Begzhan Kalemshariv, Dina Khamitova, Indira Temirova, Saule Saduakhasova, Cemile Yılmaz, Neslihan Göncüoğlu Taş, Vural Gökmen, Gulnazym Ospankulova

**Affiliations:** 1 Saken Seifullin Kazakh Agrotechnical University, Zhenis Avenue, 62, Astana 010000, Kazakhstan; kamanovasveta@mail.ru (S.K.); gaukharmadok@gmail.com (G.A.); begjan.ae@gmail.com (B.K.); dina.khamitova@nu.edu.kz (D.K.); indira_t85@mail.ru (I.T.); saulesaduahasova5@gmail.com (S.S.); 2 Aron R&D Co. LLP, Akmeshit Street, 5, Astana 010000, Kazakhstan; 3 Department of Biology, School of Sciences and Humanities, Nazarbayev University, Astana 010000, Kazakhstan; 4 LLP “Regional Technological Park QazAgroOrganic” Zhenis Avenue, 62B, Astana 010000, Kazakhstan; 5 Food Quality and Safety (FoQuS) Research Group, Department of Food Engineering, Hacettepe University, Ankara 06800, Turkey

**Keywords:** triticale, naked oat, amaranth, neuromodulators, amino acid content, antioxidant activity, GABA, aroma compounds

## Abstract

This study investigated non-conventional crops as raw materials for plant-based fermented functional products with potential neuromodulatory properties, assessing antioxidant activity, amino acid composition, neuroactive metabolites, and aroma profile. Antioxidant activity depended not only on total phenolic content but also on the composition and extractability of other bioactive and reducing constituents released during processing. Free amino acid and γ-aminobutyric acid (GABA) profiles differed markedly from the raw grains, reflecting the effects of enzymatic hydrolysis, formulation, and microbial fermentation. The naked oat fermented product (NOFP) had the highest total, essential and branched-chain free amino acid contents and contained serotonin (811 µg/kg). The triticale fermented product (TFP) showed high phenylethylamine (440 µg/kg), proline and glutamine, and the highest diacetyl content (502 ng/g), potentially contributing to its nutritional and sensory properties. The amaranth fermented product (AFP) was distinguished by high GABA (648.9 mg/kg DW) and L-DOPA (15,272 µg/kg). Kynurenine, kynurenic acid, indole-3-lactic acid, tryptamine, and other tryptophan and tyrosine metabolites were also detected. Volatile profiles were strongly matrix-dependent; AFP was the most diverse, with high levels of aldehydes, pyrazines, furans, and sulfur-containing compounds. Overall, the grain matrix and the processing and formulation sequence strongly influence the nutritional, antioxidant, neuroactive, and aroma characteristics of the products.

## 1. Introduction

In the context of the projected increase in the world’s population to 9.7 billion people by 2050, a significant rise in the global demand for food resources is expected, [1] including dietary protein—an essential macronutrient critical for meeting human physiological requirements and widely used in the food industry. In this regard, sustained growth in consumer and industry interest in dairy alternatives has been observed. Plant-based fermented products can be produced from a wide range of plant raw materials, including legumes, nuts, cereals, and other crops [2]. Among cereals, oats are widely used to produce plant-based beverages [3] as they are a good source of unsaturated fatty acids, antioxidants, and high-quality proteins [4]. Moreover, the positive effect of dietary fiber is well known, as its high β-glucan content contributes to the reduction of blood cholesterol levels [5]. Oat-based beverages are characterized by pronounced emulsifying and gel-forming properties, contributing to the formation of dense structured systems with high water-holding capacity, which is associated with their β-glucan content [6]. Owing to all these advantages, oats are widely used to produce fermented products with functional properties [7]. Similarly, the grain of the pseudocereal amaranth has a high content of sulfur-containing amino acids and lysine [8]. Furthermore, amaranth protein could absorb water and oil during gel formation and can maintain an emulsion over time, preventing coalescence and flocculation of oil globules [9,10]. In terms of its major nutrient composition, triticale grain is more similar to wheat than to rye. It is characterized by high levels of carbohydrates, protein, and dietary fiber, as well as phenolic compounds, flavonoids, vitamins, and polysaccharides, which determine its antioxidant and functional properties [11].

Fermentation increases the bioavailability of phenolic compounds, enriches them with vitamins and bioactive peptides, and reduces the content of phytates, tannins, and oxalates, thereby improving both nutritional value and digestibility [12]. Moreover, the complex interaction of proteins, polyphenols, and polysaccharides in cereal matrices contributes to the formation of taste sensations and sensory perception [13]. The formation of the neuroactive profile of cereal crops and their processed products is related to the amino acid composition of the raw material and microbial transformations, including fermentation [14,15]. The amino acids glutamic acid, tryptophan, tyrosine, phenylalanine, histidine, arginine, and glycine are central to the regulation of the nervous system and stress adaptation. Glutamic acid serves as a precursor of γ-aminobutyric acid (GABA), the primary inhibitory neurotransmitter of the central nervous system (CNS), while tryptophan is involved in the synthesis of serotonin and melatonin, which regulate mood, sleep, and adaptive responses [16,17]. Tyrosine and phenylalanine are precursors to catecholamines (dopamine and norepinephrine), which influence cognitive functions and behavioral responses, while microbial fermentation of these amino acids can lead to the formation of biogenic amines [18,19]. Histidine is a precursor of histamine, which modulates neural and immune processes, while arginine contributes to the synthesis of nitric oxide, important for neurovascular regulation [20]. Glycine acts as an inhibitory neurotransmitter, ensuring the balance of excitation and inhibition in the CNS [21]. These amino acids are of interest as functional components of plant-based and fermented products that contribute to the protection of the body against the effects of stress and support safe nutrition.

During the fermentation process, lactic acid bacteria possess the potential to convert food ingredients into aromatic compounds such as organic acids, alcohols, ketones, and others [22]. For example, certain strains of *Lactiplantibacillus plantarum* may contribute to the formation of aromatic compounds associated with aspartic acid [23], while strains of *Streptococcus thermophilus* may promote the formation of aromatic amino acid compounds [24]. It is known that aldehydes are formed during the degradation of fatty acids; for example, hexanal, a degradation product of linoleic acid, is characterized by a green and woody aroma [25]. Ketones, similarly to aldehydes, are products of the oxidation of fatty acids and linoleic acid, imparting floral, creamy, and yogurt-like aromas to products [26]. Thus, fermentation by lactic acid bacteria plays an important role in the formation of the aromatic profile of fermented products, influencing their organoleptic characteristics and consumer properties.

The aim of this study was to investigate the use of triticale grain, naked oats, and amaranth to produce fermented functional products with potential neuromodulatory properties, through a comprehensive assessment of their antioxidant activity, amino acid composition, neuroactive metabolites, and aroma profile.

## 2. Materials and Methods

### 2.1. Materials

Triticale grain (TG) was provided by the North Kazakhstan Agricultural Experimental Station LLP, naked oat grain (NOG) by the A.I. Barayev Scientific and Production Center for Grain Farming LLP, and amaranth grain (AG) was purchased from farmers in Petropavlovsk, Kazakhstan. Pea protein isolate was purchased from Partner-M JSC (Moscow, Russia). Refined deodorized rapeseed oil, grade “P”, was manufactured by Agriproduct LLC (Oberovshchina, Republic of Belarus). The vitamin premix (DEL’AR^®^) and complex food supplement “Mineral” (DEL’AR^®^) were purchased on the CIS market (Almaty, Kazakhstan). Lyophilized direct-to-vat starter cultures “Golden Time YO 22.10 VEG Golden Line (50EA)”, consisting of *Streptococcus salivarius* subsp. *thermophilus* and *Lactobacillus delbrueckii* subsp. *bulgaricus*, were purchased from Cis market (Almaty, Kazakhstan). Enzymes were purchased from Novozymes (Beijing, China).

### 2.2. Chemicals and Consumables

All chemical reagents for analyses were purchased from Sigma-Aldrich (Burlington, VT, USA).

### 2.3. Preparation of Plant-Based Fermented Products

Triticale, naked oat, and amaranth grain were used as the raw materials for the fermented product. The grains were cleaned, washed, and then pre-soaked for 2 h. The soaked grains were transferred to a food-grade reactor, mixed with water at a grain-to-water ratio of 1:5, and wet-milled under continuous stirring until a homogeneous suspension was obtained. The resulting suspension was subjected to enzymatic hydrolysis using α-amylase BAN 480 L at a dosage corresponding to 0.60 KNU-B/g of grain substrate. Hydrolysis was carried out at 65 °C for 1 h at pH 6.5. Subsequently, prior to the addition of Alcalase^®^ 2.4 L FG at a dosage of 0.0281 AU-A/g of grain substrate, the pH of the suspension was adjusted to 8.0. Proteolytic hydrolysis was then performed at 55 °C for 1 h. The enzymes were inactivated by heating the suspension at 90 °C for 10 min.

The resulting hydrolysate was centrifuged using a CM-7S Plus centrifuge (ELMI, Riga, Latvia) at 1000× *g* for 15 min. The supernatant was subsequently fortified with a vitamin premix, mineral premix, pea protein isolate, and rapeseed oil. Prior to fermentation, the formulated mixture was pasteurized at 85 °C for 10 min and cooled to 40 °C. Submerged fermentation was employed, where the mixture was inoculated with the Golden Time YO 22.10 VEG Golden Line starter culture (50EA) at a dosage of 0.02 g/L (equivalent to 0.05 EA/L) of hydrolysate.

**Figure 1:**
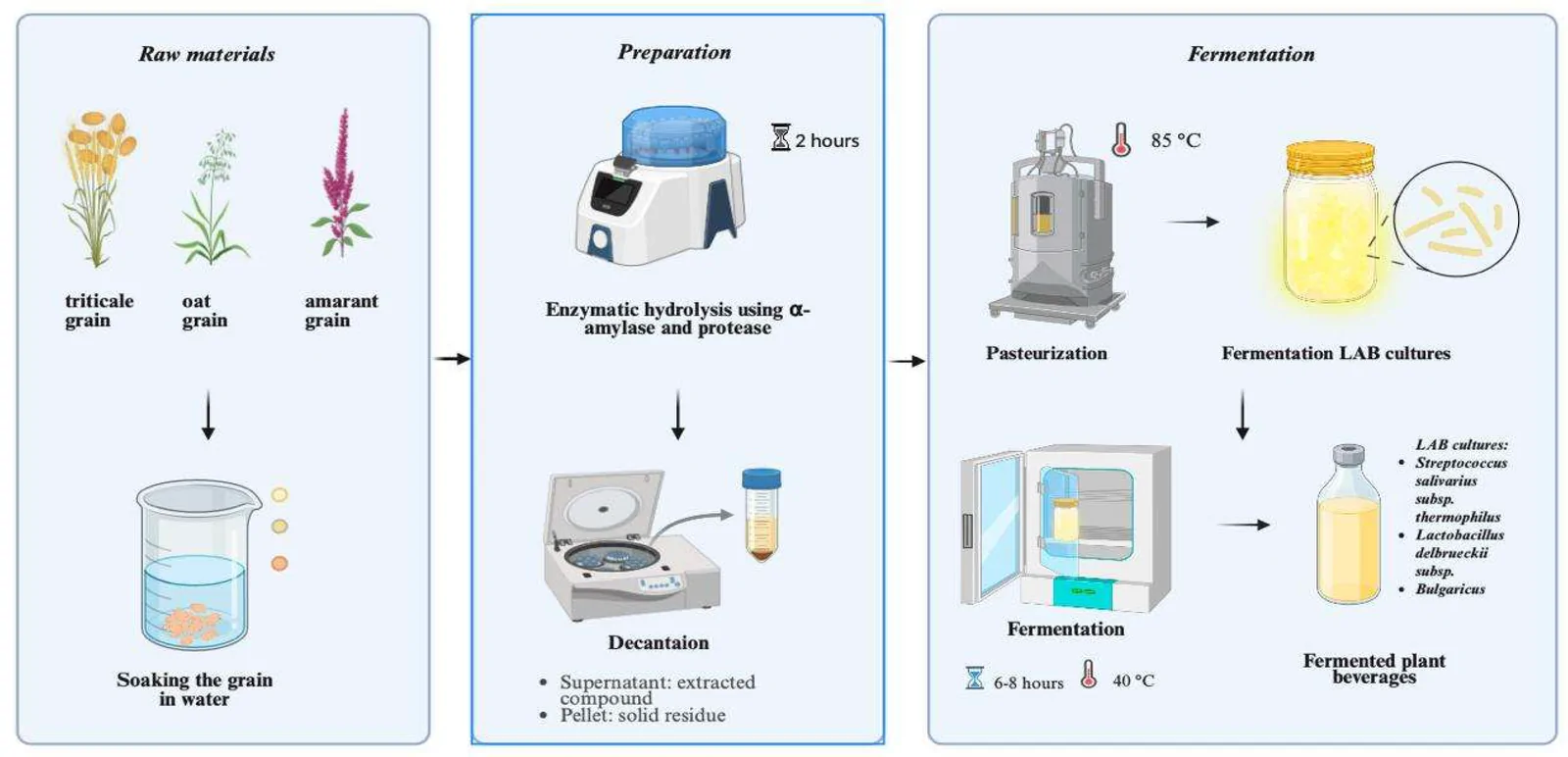
Scheme for obtaining fermented products from triticale grain, naked oats, and amaranth.

Fermentation was carried out at 40 °C for 6–8 h until a final pH of 4.7–4.8 was reached. The overall process for obtaining fermented products from triticale, naked oat, and amaranth is presented in Figure 1. Under these conditions, yogurt-like fermented products were obtained from triticale (TFP), naked oat (NOFP), and amaranth (AFP). The moisture contents of TFP, NOFP, and AFP were 75.7%, 73.6%, and 77.8%, respectively. The finished products were stored at 4 °C.

The levels of the fortifying ingredients presented in Table 1 were selected based on the composition of the respective grain hydrolysates and the technological requirements for obtaining stable, yogurt-like fermented products.

Pea protein isolate was used to complement the protein fraction naturally present in the triticale, naked oat, and amaranth hydrolysates. Its amount was adjusted individually according to the initial protein content of each hydrolysate. Similarly, the amount of refined rapeseed oil was tailored to the endogenous lipid content of each plant matrix to provide an appropriate lipid phase in the final formulation. Sunflower lecithin was added at 0.4 g/100 mL as an emulsifying agent to facilitate the formation and stabilization of the oil-in-water emulsion. A stabilizer–emulsifier blend containing mono- and diglycerides of fatty acids (E471), guar gum (E412), and xanthan gum (E415) was incorporated at 0.55 g/100 mL to improve the physical stability, viscosity, and resistance to phase separation of the final fermented products. The vitamin premix was added at 0.019 g/100 mL, and the mineral premix was incorporated at 0.165 g/100 mL, in accordance with the manufacturer-recommended dosages for beverages and fermented products. Ultimately, this standardized formulation strategy was used to minimize differences arising from the initial macronutrient composition of the raw materials, yielding nutritionally enriched, physically stable plant-based matrices suitable for subsequent fermentation and analytical evaluation.

### 2.4. Determination of Physicochemical Parameters

Before analysis, fermented product samples were lyophilized to constant weight using a HyperCool 3110 freeze-dryer (Hanil, Gimpo, Republic of Korea) operated at −50 °C under a vacuum of 0.05 mbar. The dried samples were subsequently pulverized into a uniform powder and kept at −20 °C until further use.

#### 2.4.1. Determination of Protein Content

The determination of the mass fraction of total nitrogen contained in the sample was carried out using the Kjeldahl method [27] by decomposing 1 g of the analyzed sample with sulfuric acid to form ammonium salts, converting ammonium to ammonia by alkalinization, distilling it, and determining its amount by titration.

#### 2.4.2. Determination of Fat Content

The total fat content was determined according to the method in [28]. Fat was determined by weighing 3 g of sample and extracting it with petroleum ether in a SOXTHERM SOX414 apparatus, Gerhardt (Königswinter, Germany), for 6 h.

#### 2.4.3. Determination of Ash Content

Ash content was determined according to [29]. The ash content was determined by weighing 2 g of the sample into a porcelain crucible and incinerating it at 600 °C for 6 h in a muffle furnace (SnolTherm, Narkūnai, Lithuania) until white ash was obtained. After incineration was complete, the crucibles with ash were cooled and weighed.

#### 2.4.4. Determination of Carbohydrate Content

The total carbohydrate content was determined by the anthrone-sulfuric acid colorimetric method according to [30] with modifications. Briefly, ground samples were pre-extracted. Then, 1 mL of the test sample was mixed with 4 mL of anthrone reagent in an ice bath. The resulting mixture was heated at 100 °C for 10 min, then quickly cooled to room temperature. Optical density was measured at 620 nm using a UV-1800 spectrophotometer (Shimadzu, Kyoto, Japan). Quantification was performed using a calibration curve constructed using standard glucose solutions.

#### 2.4.5. Determination of Dietary Fiber Content

An enzymatic–gravimetric approach was used to determine total dietary fiber content, following the method outlined in [31]. Briefly, samples dispersed in phosphate buffer (pH 6.0) underwent sequential treatment with α-amylase, protease, and amyloglucosidase to eliminate digestible material. The resulting insoluble fraction was precipitated with ethanol and acetone, filtered to recover the residue, and dried. Ash [29] and protein [27] contents were then subtracted from the dried residue weight to yield the final dietary fiber value.

### 2.5. Determination of Antioxidant Activity

Freeze-dried samples were ground to a homogeneous powder prior to extraction. One gram of each sample was extracted with 4 mL of 80% methanol (*v*/*v*), corresponding to a sample-to-solvent ratio of 1:4 (*w*/*v*). The mixture was homogenized for 2 min and subsequently subjected to ultrasonic extraction for 30 min at room temperature under light-protected conditions. The samples were then centrifuged at 10,000× *g* for 10 min at 4 °C. The resulting supernatant was collected and used for the determination of DPPH and ABTS radical-scavenging activities and total phenolic content.

Samples for the DPPH assay were analyzed according to the method described in [32] with slight modifications. The methanolic extract (100 μL) was mixed with 100 μL of DPPH solution (0.2 mM in 95% methanol) and incubated in the dark at 37 °C for 30 min. Absorbance was measured at 517 nm, and antioxidant activity was calculated using a Trolox calibration curve and expressed as μmol Trolox equivalents per gram of dry weight (μmol TE/g DW). The ABTS radical-scavenging activity was determined using a modified method described in [33]. The ABTS radical was generated by mixing 7 mM ABTS solution with 2.6 mM potassium persulfate, followed by incubation in the dark for 24 h. To 90 µL of the ABTS working solution, 10 µL of the sample was added, and the mixture was incubated for 30 min at room temperature. Absorbance was measured at 734 nm. Antioxidant activity was calculated using a Trolox calibration curve and expressed as μmol TE/g DW.

### 2.6. Determination of Total Phenolic Content

The same methanolic extracts prepared as described in Section 2.5 were used for the determination of total phenolic content using the Folin–Ciocalteu method [34]. This method is based on the reducing capacity of the Folin–Ciocalteu reagent under alkaline conditions. Briefly, 0.5 mL of the extract was mixed with 2.5 mL of Folin–Ciocalteu reagent (0.2 mol/L) and incubated in the dark for 5 min at room temperature. Subsequently, 2.0 mL of 10% sodium carbonate solution was added, and the mixture was thoroughly mixed and allowed to stand for 1 h at room temperature. Absorbance was measured at 700 nm using a UV-1900i spectrophotometer (Shimadzu, Japan). Total phenolic content was calculated using a gallic acid calibration curve and expressed as mg gallic acid equivalents per gram of dry weight (mg GAE/g DW).

### 2.7. Determination of Amino Acid Profile

Freeze-dried and ground grain and fermented-product samples (1 g) were extracted with water containing 0.1 M HCl in three sequential steps (10, 5, and 5 mL). After mixing, they were centrifuged (3000× *g* for 5 min). The supernatant was filtered through 0.45 µm syringe filters into Eppendorf tubes. Then, 450 µL of the filtered sample, 500 µL of a mixture of acetonitrile and water (80:20, *v*/*v*), and 50 µL of an internal standard (mixture of isotopically labeled ^13^C, ^15^N amino acids) were mixed in an autosampler vial.

Amino acid analysis was performed using an ultra-high-performance liquid chromatography-tandem mass spectrometer (UPLC-MS/MS) (Waters Corp., Milford, MA, USA) according to a previously described method [35]. Chromatographic separation was carried out on a Merck ZIC-HILIC column (150 × 4.6 mm inner diameter, 3.5 µm) using a mobile phase consisting of 0.1% formic acid in water (A) and 0.1% formic acid in acetonitrile (B) at a flow rate of 1 mL/min.

The gradient program was as follows: 0–4 min, 20% A; 4–7 min, 80% A; 7–10 min, 80% A; 10–11 min, 20% A; 11–15 min, 20% A. The column temperature was 40 °C, and the injection volume was 5 µL. The mass spectrometer source was used in positive ionization mode. The electrospray source settings were as follows: cone voltage 15 V; capillary voltage 2.5 kV; source temperature 150 °C; desolvation temperature 600 °C; desolvation gas (nitrogen) flow rate 1000 L/h; and cone gas flow rate 50 L/h. Amino acids were identified by multiple reaction monitoring (MRM). Quantification was performed using an internal standard calibration method based on isotope dilution, with calibration curves constructed from the peak area ratio of each target analyte to its corresponding isotope-labeled internal standard. Calibration curves were established over the concentration range of 0.5–43.75 µM (0, 0.5, 1, 2, 5, 10, 17.5, and 43.75 µM).

### 2.8. Determination of Neuroactive Compound Profile

Freeze-dried and ground samples (0.5 g) were extracted with water in three sequential steps (5, 2.5, and 2.5 mL). After vortex mixing for 3 min and centrifugation at 3000× *g* for 5 min at each extraction step, the supernatants were combined. The combined extracts were stored at −80 °C for 30 min to precipitate proteins. The supernatant was then diluted 2-fold with water. After that, the extracts were filtered through a 0.45 µm syringe filter into an autosampler vial.

Analysis of amino acid compounds was performed using an ultra-high-performance liquid chromatography-tandem mass spectrometer (UPLC-MS/MS) (Waters Corp., Milford, MA, USA) according to a previously described method [36]. Chromatographic separation was carried out on an Accucore Phenyl-Hexyl column (150 × 4.6 mm inner diameter, 2.6 µm) using a combination of 0.2% formic acid in water (A) and 0.1% formic acid in methanol (B) at a flow rate of 0.7 mL/min. The gradient program was as follows: 0–3 min, 90% A; 3–10 min, 10% A; 10–11 min, 10% A; 11–12 min, 90% A; 12–15 min, 90% A. The column temperature was 30 °C, and the injection volume was 5 µL. The MS source was used in positive ionization mode. The electrospray source settings were as follows: cone voltage 20 V; capillary voltage 1.0 kV; extractor voltage 2 V; source temperature 130 °C; desolvation temperature 450 °C; desolvation gas (nitrogen) flow rate 600 L/h; and cone gas flow rate 50 L/h. Amino acid compounds were identified by MRM, and quantification was performed using external calibration curves constructed for all amino acid compounds in the range from 1 to 1000 µg/L (1–2–5–10–20–50–100–200–500–1000 µg/L).

### 2.9. Determination of Volatile Compounds Profile

For analysis, 2 g of freeze-dried and ground sample was placed into a 20 mL headspace vial. Isopropylpyrazine (0.05 ppm) was used as the internal standard for pyrazines, whereas 3-methyl-2-butanone (0.5 ppm) was used as the internal standard for all other quantified volatile compounds. Extraction was carried out using the SPME method employing a Thermo TriPlus RSH autosampler. The vial was preheated at 60 °C for 10 min, and the extraction of volatile compounds was performed at 60 °C for 30 min using a CAR/PDMS/DVB SPME-Arrow (Thermo Fisher Scientific, Waltham, MA, USA).

Desorption was carried out at 230 °C for 1 min using a Thermo Trace 1300 GC + Thermo ISQ MS system (Thermo Fisher Scientific, Waltham, MA, USA). Sample introduction was performed in splitless mode: carrier gas was helium, 1.2 mL/min, with an InertCap WAX column, 60 m × 0.25 mm × 0.25 μm. The temperature was held at 30 °C for 5 min, then increased to 230 °C at a rate of 5 °C/min, and the process was held for 10 min. Ionization was performed using EI at 70 eV, with a source temperature of 230 °C, and the scanning range was *m*/*z* 35–400. Linear retention indices (LRIs) of the volatile compounds were calculated using an n-alkane standard mixture (C7–C30 in hexane) (Sigma-Aldrich, St.Louis, MO, USA) analysed under the same chromatographic conditions. Comparison of mass spectra and LRI values was performed with authentic reference standards using the Wiley 9 and NIST 21 libraries. Compounds without standards were tentatively identified based on LRI values in the literature [37].

### 2.10. Statistical Analysis

In the study, statistical data processing was performed using analysis of variance (ANOVA) with Tukey’s (HSD) test to compare amino acid levels in the samples and identify significant differences. The analysis was carried out using the statistical software package Minitab (version 22) [38]. The bar chart of antioxidant activity and polyphenol content was built in SPSS Statistics software version 31.0 (IBM, Armonk, NY, USA), and the heat map with hierarchical clustering was generated in RStudio version 2025.09 (Posit Software, Boston, MA, USA). Results are presented as mean ± standard deviation (SD). Statistical significance was set at *p* < 0.05. All experiments were performed in independent triplicates.

## 3. Results

### 3.1. Physicochemical Properties

The nutritional value of fermented plant-based products depends on the raw materials. The use of cereal crops results in high fiber and carbohydrate content, whereas the use of legumes and pseudocereals provides high protein content [4,39].

The chemical composition of TG, NOG, and AG, together with the corresponding final fermented products (TFP, NOFP, and AFP), is presented in Table 2. To allow a more appropriate comparison between the dry grain materials and the high-moisture fermented products, all compositional data were expressed on a dry weight basis (DW). This normalization eliminates the direct effect of differences in moisture content and allows the relative distribution of the major dry-matter constituents to be compared among the samples.

The raw grains differed significantly in their macronutrient composition. NOG showed the highest protein content (18.2% DW), whereas AG and TG contained 14.8% and 14.2% DW, respectively. Carbohydrates were the major fraction in TG (71.7% DW) and AG (70.2% DW), with a lower value in NOG (59.6% DW). NOG and AG had higher lipid contents (6.1% and 6.5% DW, respectively) than TG (1.9% DW). Dietary fiber was highest in NOG (8.3% DW), followed by AG (4.2% DW) and TG (3.5% DW).

The composition of the final fermented products differed markedly from that of the corresponding raw grains. These differences should not be interpreted as a direct effect of fermentation alone: the production process included soaking, wet milling, enzymatic hydrolysis, centrifugation, and fortification with pea protein isolate, rapeseed oil, and vitamin and mineral premixes, prior to lactic acid fermentation. The composition of TFP, NOFP, and AFP therefore reflects the cumulative effect of this entire sequence, consistent with reports that processing and formulation steps can substantially shape the final composition of plant-based fermented products [40,41].

Among the fermented products, protein content ranged from 11.4 to 13.5% DW, highest in AFP (13.5%), followed by TFP (12.4%) and NOFP (11.4%). Despite the addition of pea protein isolate, the relative protein proportion was lower than in the corresponding raw grains—not evidence of protein degradation, but a reflection of dry-matter redistribution following the addition of lipid and mineral components and the partial removal of insoluble material during centrifugation.

Fat content showed the most pronounced change, ranging from 14.0 to 16.7% DW and significantly exceeding that of the raw grains, highest in AFP (16.7%), followed by TFP (15.2%) and NOFP (14.0%). This reflects the formulation strategy, in which rapeseed oil was added according to the endogenous lipid content of each hydrolysate to standardize the fat phase, producing formulation-driven differences among the three products.

Carbohydrates remained the largest individual fraction (32.9–39.9% DW) but were proportionally lower than in the raw grains, highest in TFP (39.9%) and similar in NOFP and AFP (32.9% and 33.8%). This likely reflects several concurrent factors: starch conversion by α-amylase, partial removal of insoluble carbohydrate material during centrifugation, fermentable sugar utilization during fermentation, and dilution by the added protein, lipid, and mineral components. As intermediate samples were not analyzed, the relative contribution of each mechanism cannot be distinguished.

Ash content was likewise substantially higher in the fermented products (7.5–10.4% DW) than in the raw grains (2.1–3.9% DW), highest in AFP (10.4%), followed by TFP (7.8%) and NOFP (7.6%), consistent with the incorporation of the mineral premix, though the final value reflects the overall mineral fraction of the formulated product rather than the premix alone.

Dietary fiber ranged from 6.8 to 7.5% DW in the fermented products, highest in NOFP (7.5%), followed by TFP (7.0%) and AFP (6.8%), compared with a maximum of 8.3% DW in NOG. These differences likely reflect both the separation of insoluble fractions during processing and shifts in the relative proportions of other dry-matter constituents, and should be read as characteristics of the final formulated matrix rather than a fermentation-specific effect.

Overall, expressing results on a dry weight basis shows that the final composition of the fermented products was shaped by both the intrinsic grain matrix and the overall processing strategy: the elevated lipid and ash content primarily reflects formulation, while the lower relative contribution of carbohydrates and protein reflects dry-matter redistribution during hydrolysis, centrifugation, fortification, and fermentation. The differences between raw grains and fermented products are therefore best understood as compositional differences between two distinct matrices, not as direct metabolic effects of lactic acid fermentation alone.

### 3.2. Total Antioxidant Activity and Total Phenolic Content

**Figure 2:**
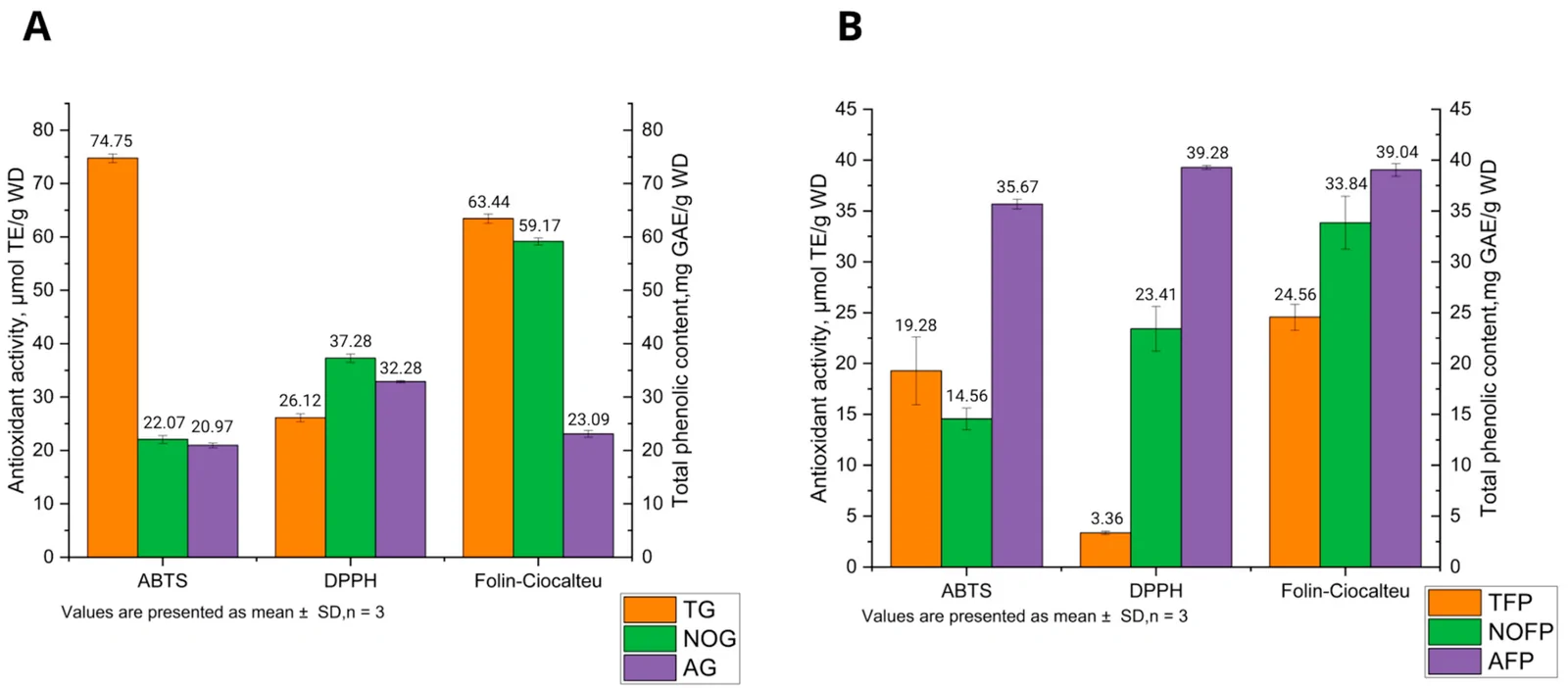
Total phenolic content and antioxidant activity of raw grains and corresponding fermented products. (**A**) Raw grains: triticale grain (TG), naked oat grain (NOG), and amaranth grain (AG); (**B**) fermented products: triticale fermented product (TFP), naked oat fermented product (NOFP), and amaranth fermented product (AFP). Antioxidant activity was determined using the ABTS and DPPH assays, and total phenolic content was determined using the Folin–Ciocalteu method. Values are expressed as mean ± SD (*n* = 3) on a dry weight basis (DW).

The antioxidant activity of food products is critical for extending shelf life and preventing oxidative damage in the human body [42]. The total phenolic content (TPC) and antioxidant activity of the raw grains and the corresponding fermented products are presented in Figure 2.

The results revealed a pronounced matrix-dependent response to processing. Among the raw grains, TG exhibited the highest total phenolic content (63.44 mg GAE/g DW) and ABTS radical-scavenging activity (74.75 µmol TE/g DW), whereas NOG showed the highest DPPH activity (37.28 µmol TE/g DW). AG had the lowest total phenolic content (23.09 mg GAE/g DW), with DPPH and ABTS activities of 32.88 and 20.97 µmol TE/g DW, respectively.

The triticale-based system showed the most pronounced decrease after processing: TFP’s total phenolic content fell from 63.44 to 24.56 mg GAE/g DW (≈61%), DPPH activity from 26.12 to 3.36 µmol TE/g DW (≈87%), and ABTS activity from 74.75 to 19.28 µmol TE/g DW (≈74%). A similar but less pronounced pattern occurred in the naked oat system: TPC decreased from 59.17 to 33.84 mg GAE/g DW (≈43%), DPPH from 37.28 to 23.41 µmol TE/g DW (≈37%), and ABTS from 22.07 to 14.56 µmol TE/g DW (≈34%).

The amaranth-based system showed the opposite trend: AFP’s TPC rose from 23.09 to 39.04 mg GAE/g DW (≈69%), accompanied by increases in DPPH (32.88 to 39.28 µmol TE/g DW, ≈19%) and ABTS (20.97 to 35.67 µmol TE/g DW, ≈70%) activity, suggesting that changes in Folin–Ciocalteu-reactive compounds contributed to the enhanced antioxidant capacity of this product.

Across all three systems, the direction of change in TPC was consistent with the direction of change in DPPH and ABTS activity, though the magnitude differed between assays—indicating that antioxidant activity was not determined by phenolic content alone, but also by the composition, extractability, and radical-specific reactivity of phenolic compounds and other reducing constituents. These differences cannot be attributed exclusively to fermentation: the production process also included soaking, wet milling, enzymatic hydrolysis, centrifugation, fortification, and pasteurization, any of which may have altered the extractability or removed phenolic-containing insoluble fractions. The antioxidant characteristics of TFP, NOFP, and AFP should therefore be read as the cumulative outcome of this full sequence, consistent with reports that probiotic fermentation alone does not produce a uniform antioxidant response across plant matrices [43].

Several non-phenolic mechanisms likely contributed alongside phenolic compounds. Enzymatic hydrolysis releases free amino acids and short peptides from grain proteins. Residues such as methionine, tyrosine, tryptophan, and proline, along with hydrophobic, aromatic, or sulfur-containing peptides, can contribute modest radical-scavenging activity, complementary to rather than a substitute for phenolic antioxidants. This is consistent with NOFP and TFP, where such compounds were not sufficient to offset the overall decrease in DPPH and ABTS activity relative to the raw grains. Low-molecular-weight metabolites may also have played a role: L-DOPA, detected in AFP (15272 µg/kg) and NOFP (9235 µg/kg), can participate in hydrogen- and single-electron-transfer reactions via its catechol structure, and indole-3-lactic acid, detected in both products, has an electron-rich indole ring associated with antioxidant activity [44]. However, NOFP’s DPPH and ABTS activities remained lower than NOG’s despite these metabolites, indicating that processing-related losses in phenolic extractability outweighed any contribution from them. Similarly, diacetyl was reported to show DPPH-scavenging activity [45] and was detected at its highest level in TFP (502 ng/g), yet TFP had the lowest DPPH activity of all three products (3.36 µmol TE/g DW), confirming that none of these minor constituents was a dominant driver of antioxidant capacity.

Overall, the antioxidant characteristics of the fermented products reflect a matrix-dependent balance between the loss, transformation, and release of native phenolic compounds and the contribution of non-phenolic reducing constituents generated during processing. Because intermediate samples were not analyzed after each technological stage, the individual contributions of hydrolysis, centrifugation, fortification, pasteurization, and fermentation cannot be separated—the observed antioxidant profiles should be interpreted as the cumulative outcome of the complete processing and formulation sequence rather than a direct effect of fermentation alone.

### 3.3. Free Amino Acids Profile

Fermentation by lactic acid bacteria promotes an increase in the concentrations of free amino acids and peptides, soluble fiber, and total phenols, which corresponds to higher protein digestibility and improved nutritional value of the fermented product [46]. Biological acidification is also associated with a reduction in the starch hydrolysis index, primarily through the formation of resistant starch, which lowers the glycemic index of the fermented product [47].

The free amino acid profiles differed markedly among the three final fermented products. It should be emphasized that the production process included soaking, wet milling, enzymatic treatment with α-amylase and protease, centrifugation, fortification with pea protein isolate, and subsequent lactic acid fermentation. Therefore, differences between the raw grains and the fermented product cannot be attributed specifically to fermentation and should be interpreted as the combined outcome of raw-material composition and the entire processing sequence. The results for the content of free amino acids in raw grain and fermented products are given in Table 3.

Comparative analysis of TG and TFP revealed substantial differences in free amino acid content. The total amount of free amino acids and GABA was approximately 3.5-fold higher in TFP than in TG—a difference attributable to the cumulative effect of enzymatic hydrolysis, centrifugation, formulation, and fermentation rather than fermentation alone. Histidine increased from 4.8 to 69.6 mg/kg (14.5-fold) and lysine from 8.5 to 150.0 mg/kg (17.6-fold), while tryptophan was lower in TFP than in TG (70.5 vs. 219.1 mg/kg, a 3.1-fold decrease). Although microbial utilization of tryptophan during fermentation has been reported elsewhere [48,49], this cannot be confirmed here, as intermediate samples were not analyzed.

The higher free amino acid levels likely reflect the combined effects of proteolysis, matrix changes during processing, fortification, and fermentation. Total essential amino acids in TFP reached 1905.4 mg/kg, approximately 2.6-fold higher than the 725.7 mg/kg in TG. Among non-essential amino acids, TFP showed a notably high proline content (800.5 vs. 184.0 mg/kg in TG), likely reflecting the proline-rich fractions of the triticale protein complex [50], which are highly susceptible to the applied proteases. Glutamine (635.9 vs. 99.3 mg/kg), serine (319.0 vs. 95.2 mg/kg), and alanine (384.0 vs. 199.7 mg/kg) were similarly elevated, as was GABA (571.9 vs. 29.8 mg/kg in TG).

The largest overall difference between raw grain and final product was observed in the naked oat system: total free amino acids and GABA reached 9457.1 mg/kg in NOFP versus 1395.6 mg/kg in NOG (≈6.8-fold). The most pronounced shifts occurred among non-essential and conditionally essential amino acids—arginine (809.7 vs. 2.7 mg/kg, ~300-fold), lysine (341.0 vs. 3.4 mg/kg, ~100-fold), and histidine (149.8 vs. 2.5 mg/kg, ~60-fold)—consistent with reports that lactic acid bacterial bioprocessing can alter amino acid availability [51], though this contribution cannot be separated here from that of hydrolysis and formulation.

This pattern is consistent with extensive proteolysis of naked oat proteins during enzymatic pretreatment. Essential amino acids in NOFP showed marked increases over the raw grain, including methionine (~28-fold), leucine (~13-fold), and threonine (~7-fold), with total essential amino acids reaching 4762.5 mg/kg versus 594.3 mg/kg (~8-fold). Leucine alone reached 1330.9 mg/kg, contributing to a high total BCAA content (isoleucine + valine + leucine) of 2274.3 mg/kg in NOFP versus 311.0 mg/kg in NOG. This is a nutritionally relevant increase, as BCAAs are key substrates for skeletal muscle energy metabolism and post-exercise recovery [52].

Asparagine was lower in NOFP than in NOG (130.3 vs. 181.0 mg/kg), and tryptophan was, unlike in the triticale and amaranth systems, higher rather than lower (350 vs. 86.3 mg/kg). Microbial utilization of asparagine during fermentation has been reported previously [53], but as with tryptophan, this cannot be attributed to a specific mechanism here, since intermediate processing stages were not analyzed.

In the amaranth system, AFP also showed a distinct amino acid profile relative to AG, though the pattern differed from the triticale and naked oat matrices. The most striking difference was in asparagine (670.3 vs. 14.0 mg/kg, 48-fold), alongside higher lysine (171.9 vs. 3.9 mg/kg) and histidine (55.3 vs. 2.35 mg/kg). Glycine was undetected in free form in AG—where it is largely bound in storage proteins [54]—but reached 78.7 mg/kg in AFP.

AFP was further characterized by relatively high lysine (171.9 mg/kg), aspartic acid (169.5 mg/kg), and arginine (343.9 mg/kg), likely reflecting the added pea protein isolate as an additional source of these amino acids. Conversely, isoleucine (88.0 vs. 122.2 mg/kg), tryptophan (77.9 vs. 328.0 mg/kg), and glutamine (95.5 vs. 134.1 mg/kg) were lower than in AG—a shift that may reflect multiple processing-related transformations rather than microbial utilization alone [44,55,56]. GABA was substantially higher in AFP than in AG (648.9 vs. 33.4 mg/kg), one of the largest compositional shifts in this system, and nearly comparable to the GABA level in NOFP.

Overall, each grain–product pair showed distinct, matrix-dependent shifts in free amino acid and GABA profiles, reflecting the combined effect of enzymatic hydrolysis, formulation, and fermentation rather than any single processing stage. These shifts were most pronounced in the naked oat system, followed by triticale and amaranth. The processed triticale product is characterized by high proline and glutamic acid, the naked oat product by the highest total free amino acid and BCAA content, and the amaranth product by elevated asparagine alongside high GABA. Glutamic acid and proline support protein metabolism, essential amino acids contribute to dietary protein quality, and GABA acts as a bioactive neuromodulator, so this combined processing approach may enhance the nutritional and functional value of the fermented products. This is consistent with broader evidence that multi-stage biochemical breakdown, combining enzymatic hydrolysis and microbial metabolism, liberates free amino acid pools and promotes the formation of bioactive metabolites such as GABA and tryptophan-related compounds [57].

### 3.4. Neuroactive Compounds Profile

Products fermented by lactic acid bacteria may contain certain neuroactive compounds that affect human health and mood [19]. The profile of detected neuroactive compounds in fermented food products is presented in Table 4.

The final fermented products contained various biogenic amines and amino acid-related metabolites.

Compared to the other fermented products studied, TFP was distinguished by a high phenylethylamine content (440 µg/kg). Phenylethylamine exerts psychoactive effects and is capable of modulating mood [58]. The histamine content in TFP was 2026 µg/kg; L-Dopa and tyramine were 1113 µg/kg and 1221 µg/kg, respectively. Histamine and tyramine are biogenic amines that may participate in the regulation of vascular tone and neurotransmission [59]. L-DOPA is a dopamine precursor and may support cognitive function and motor regulation [60]. The kynurenine content was 309 µg/kg, while kynurenic acid was detected at 45 µg/kg. Serotonin, tryptamine, and indole compounds were not detected. TFP may thus be considered a functional product rich in neuroactive compounds.

The analysis revealed high serotonin content in NOFP, at 811 µg/kg, whereas it was not detected in the other products. NOFP was also distinguished by elevated levels of histamine and tyramine, at 2055 µg/kg and 1970 µg/kg, respectively. The L-DOPA and indole-3-lactic acid contents were 9235 µg/kg and 1060 µg/kg, respectively. The high L-DOPA content is of interest from a functional nutrition perspective, as it is a direct precursor of dopamine. Indole-3-lactic acid has been reported as a metabolite associated with lactic acid bacterial fermentation and possesses neuroprotective and anti-inflammatory properties [44]. The low phenylethylamine level reduces the risk of central nervous system hyperstimulation [61], thereby positioning the fermented oat product as a functional product with pronounced neuromodulatory potential. Tryptamine was not detected in NOFP, as was the case with TFP.

Among the final fermented products, AFP exhibited the highest L-DOPA concentration (15,272 µg/kg), together with tryptamine (373 µg/kg), kynurenic acid (1539 µg/kg), and indole-3-lactic acid (1372 µg/kg). This metabolite profile is consistent with the presence of compounds associated with tryptophan metabolism and the potential of the fermented product for neuroprotection and modulation of the dopaminergic system. According to current research on fermented products, tryptophan metabolites, including kynurenine and kynurenic acid, are frequently detected in yogurt, kefir, and other fermented products as a result of microbial metabolic activity [62]. The high L-DOPA content makes amaranth of interest for the development of functional products that support cognitive function and the nervous system [63].

The fermented products were therefore characterized by distinct profiles of biologically active metabolites associated with tyrosine and tryptophan metabolism.

### 3.5. Aroma Profile

The identified volatile compounds were classified into several major chemical groups: aldehydes, ketones, alcohols, acids, pyrazines, furans, sulfur-containing compounds, and lactones (Table 5). This classification is widely applied in studies of the aroma profiles of fermented products.

The aroma compound profiles of the samples studied differed substantially depending on the grain raw material used. The highest total content of quantified volatile compounds was observed in AFP, which was characterized by the most diverse aroma profile among the final products.

Aldehydes constituted one of the most abundant compound groups and played an important role in the formation of fresh, green, and fruity aroma notes. In AFP, the content of all quantified aldehydes was statistically significantly higher (*p* < 0.05). Particularly notable differences were observed for hexanal (4377 ng/g), benzaldehyde (1597 ng/g), and phenylacetaldehyde (1923 ng/g). Hexanal is a product of unsaturated fatty acid oxidation and imparts grassy and green notes to the product. Benzaldehyde and phenylacetaldehyde contribute sweet almond and floral aroma nuances. This may be related to the high lipid content and active amino acid metabolism in this product.

Ketones may originate from fatty acid and carbohydrate transformations during processing and fermentation. Particularly high 2,3-butanedione content was observed in TFP (502 ng/g). This compound imparts creamy, buttery, and dairy notes. According to the literature, diacetyl is one of the key compounds responsible for the aroma of fermented products [64]. From a dairy industry perspective, diacetyl is generally regarded as a quality marker that contributes to the natural flavor and creaminess of products [65]. Ling et al. [45] further proposed that diacetyl may effectively act as a DPPH radical scavenger, supporting its application in preservation and as an antioxidant. AFP was characterized by the highest contents of 2-heptanone and 2-nonanone, at concentrations of 240 and 9 ng/g, which contribute to fruity and cheesy aroma nuances.

Alcohols are formed through the reduction of aldehydes or amino acid metabolism. TFP contained the highest 1-pentanol content (752 ng/g), a compound responsible for fruity aroma. The highest 1-octen-3-ol level was found in NOFP at 1714 ng/g, consistent with the literature [66]. This compound imparts mushroom and earthy notes. AFP was distinguished by high concentrations of 2-phenylethanol and benzenemethanol at 348 and 320 ng/g, respectively—these compounds impart floral and honey aromas.

The highest acetic acid concentrations were found in AFP and NOFP at 2283 and 2201 ng/g, respectively. Acetic acid may arise from microbial metabolism and other processing-related transformations and contributes to flavor development. The highest concentrations of hexanoic acid and nonanoic acid were found in TFP at 5650 and 1957 ng/g, respectively.

Of particular interest is the pyrazine content, as pyrazines contribute cereal, nutty, and roasted notes. AFP substantially exceeded the other fermented products in 2,6-dimethylpyrazine content at 59 ng/g, consistent with the presence of compounds commonly associated with Maillard-type reactions. The lowest 2,5-dimethylpyrazine concentration was found in NOFP at 1 ng/g.

The highest furan concentrations were found in AFP. The 2-pentylfuran content was 1257 ng/g, nearly five times higher than in NOFP. The lowest total furan content was found in NOFP. These compounds are formed through lipid oxidation and thermal processing of raw materials, imparting caramel, nutty, and roasted notes to the products [67].

Analysis of sulfur-containing compounds revealed that AFP contained the highest amounts of methional and dimethyltrisulfide at 201 and 120 ng/g, respectively. Dimethyltrisulfide is a product of methionine degradation and imparts characteristic potato and malty notes to products. The highest dimethyldisulfide content was found in TFP at 17 ng/g. Methional, dimethyltrisulfide, and dimethyldisulfide are also frequently reported in fermented cereal products [66].

Among the lactones, AFP exhibited the highest concentrations of gamma-octalactone and gamma-nonalactone (71 and 172 ng/g, respectively), whereas the highest gamma-hexalactone content was observed in TFP (244 ng/g). These compounds may contribute creamy and coconut-like notes to the aroma profile.

The final fermented products contained a complex mixture of volatile compounds belonging to several chemical classes. Aldehydes, organic acids, alcohols, and ketones make the most substantial contributions to the aroma profiles of the samples studied. AFP was characterized by the most diverse volatile profile, which may reflect differences in the initial grain matrix and transformations occurring throughout processing and fermentation. Because volatile profiles of the raw grains or unfermented formulations were not available, the detected volatile compounds are discussed as characteristics of the final fermented products, and their formation cannot be attributed exclusively to microbial fermentation.

## 4. Conclusions

The study demonstrated that triticale, naked oat, and amaranth can serve as distinct plant matrices for developing yogurt-like fermented products with differentiated nutritional, antioxidant, neuroactive, and aroma profiles. The final characteristics of TFP, NOFP, and AFP reflected the cumulative effects of initial grain composition, enzymatic hydrolysis, centrifugation, fortification, pasteurization, and subsequent lactic acid fermentation, rather than fermentation alone.

Among the fermented products, AFP exhibited the highest total phenolic content (39.04 mg GAE/g DW) along with the highest DPPH and ABTS radical-scavenging activities (39.28 and 35.67 µmol TE/g DW, respectively). NOFP showed the highest total free amino acid content, at approximately 9457 mg/kg DW, including 4762.5 mg/kg of essential amino acids and 2274.3 mg/kg of branched-chain amino acids. High GABA levels were detected across all three products, reaching 571.9 mg/kg in TFP, 651.0 mg/kg in NOFP, and 648.9 mg/kg in AFP. TFP was further characterized by elevated levels of proline (800.5 mg/kg), glutamine (635.9 mg/kg), phenylethylamine (440 µg/kg), and diacetyl (502 ng/g). NOFP was distinguished by the presence of serotonin (811 µg/kg), while AFP contained the highest L-DOPA concentration (15,272 µg/kg).

The volatile profiles were likewise strongly matrix-dependent. AFP displayed the most diverse aroma profile, with relatively high levels of aldehydes, pyrazines, furans, and sulfur-containing compounds, whereas TFP was distinguished by its high diacetyl content. Overall, these results indicate that grain matrix selection strongly shapes the nutritional, antioxidant, neuroactive, and aroma characteristics of fermented products, and support the potential of triticale, naked oat, and amaranth for developing nutritionally enriched plant-based fermented foods. Because intermediate processing stages were not analyzed separately, the observed differences should be interpreted as the cumulative outcome of the complete processing and formulation sequence, rather than as direct effects of lactic acid fermentation alone.

## Figures and Tables

**Table 1:** Composition and functional role of additives in the fermented products.

Additive	Characteristics	Proportion Used for Fortification (g/100 mL)
Vitamin premix	Pantothenic acid (calcium D-pantothenate)—9 g, B6 (pyridoxine hydrochloride)—1.9 g, B1 (thiamine hydrochloride)—1.45 g, Folacin (folic acid)—0.27 g, B12 (cyanocobalamin)—1 mg, Ascorbic acid (Vitamin C)—61 g, Niacin (nicotinamide)—15.7 g.	0.019
Complex food supplement “Mineral”	Premix for an isotonic-flavored beverage. Content at the recommended dosage per 100 mL: Sodium—51 mg, Potassium—13 mg, Magnesium—0.6 mg, Calcium—1.4 mg.	0.165
Pea protein	Pea protein isolate. Nutritional and energy value per 100 g: Protein—82 g, Fats—4 g, Carbohydrates—3 g, Energy—380 kCal.	depending on the type of grain raw material used
Lecithin	Sunflower lecithin, liquid, refined, E322.	0.4
Rapeseed oil	Refined deodorized rapeseed oil	depending on the type of grain raw material used
Stabilizer mix	Food-grade stabilizer–emulsifier blend containing mono- and diglycerides of fatty acids (E471), guar gum (E412), and xanthan gum (E415).	0.55

Note: Proportions are expressed as g additive/100 mL product. The amounts of pea protein isolate and rapeseed oil were not fixed across the three matrices but were adjusted individually for each grain hydrolysate (triticale, naked oat, amaranth) to compensate for differences in their endogenous protein and lipid content.

**Table 2:** Chemical composition of raw grains and fermented products on a dry weight basis (% DW).

Component	TG	TFP	NOG	NOFP	AG	AFP
Protein	14.2 ± 0.32 ^b,c^	12.4 ± 0.33 ^d^	18.2 ± 0.46 ^a^	11.4 ± 0.54 ^d^	14.8 ± 0.15 ^b^	13.5 ± 0.33 ^c^
Fat	1.9 ± 0.60 ^e^	15.2 ± 0.23 ^b^	6.1 ± 0.60 ^d^	14.0 ± 0.05 ^c^	6.5 ± 0.26 ^d^	16.7 ± 0.30 ^a^
Carbohydrates	71.7 ± 2.52 ^a^	39.9 ± 0.99 ^c^	59.6 ± 1.70 ^b^	32.9 ± 0.95 ^d^	70.2 ± 3.90 ^a^	33.8 ± 1.20 ^d^
Ash	2.1 ± 0.35 ^d^	7.8 ± 0.36 ^b^	2.4 ± 0.35 ^d^	7.6 ± 0.15 ^b^	3.9 ± 0.80 ^c^	10.4 ± 0.40 ^a^
Dietary fiber	3.5 ± 0.55 ^c^	7.0 ± 0.62 ^b^	8.3 ± 0.35 ^a^	7.5 ± 0.25 ^a,b^	4.2 ± 0.50 ^c^	6.8 ± 0.38 ^b^

Note: Values are expressed as mean ± standard deviation (*n* = 3) on a dry weight basis (DW). Different superscript letters within the same row indicate statistically significant differences among samples (*p* < 0.05), according to one-way ANOVA followed by Tukey’s HSD test.

**Table 3:** Free amino acid profile for grain and fermented product.

Amino Acids	Amount (mg kg^−1^ DW)					
	TG	TFP	NOG	NOFP	AG	AFP
Alanine	199.7 ± 8.25 ^d^	384.0 ± 1.16 ^b^	94.7 ± 3.22 ^e^	689.8 ± 0.17 ^a^	98.3 ± 2.76 ^e^	321.5 ± 4.17 ^c^
Arginine	16.7 ± 1.24 ^d^	520.8 ± 8.88 ^b^	2.7 ± 0.04 ^d,e^	809.7 ± 8.95 ^a^	0.5 ± 0.02 ^e^	343.9 ± 6.42 ^c^
Aspartic acid	74.7 ± 2.08 ^d^	278.6 ± 7.00 ^a^	23.0 ± 1.53 ^e^	284.0 ± 8.34 ^a^	130.4 ± 9.02 ^c^	169.5 ± 7.80 ^b^
Asparagine	286.7 ± 2.07 ^c^	471.6 ± 3.80 ^b^	181.0 ± 1.53 ^d^	130.3 ± 5.53 ^e^	14.0 ± 1.54 ^f^	670.3 ± 3.87 ^a^
GABA	29.8 ± 1.91 ^d^	571.9 ± 1.83 ^b^	42.6 ± 2.81 ^c^	651.0 ± 8.09 ^a^	33.4 ± 2.67 ^c,d^	648.9 ± 2.96 ^a^
Glutamic acid	171.9 ± 7.04 ^c^	540.0 ± 8.98 ^a^	89.1 ± 10.00 ^e^	423.7 ± 2.70 ^b^	103.6 ± 4.11 ^e^	134.4 ± 7.72 ^d^
Glutamine	99.3 ± 3.03 ^d,e^	635.9 ± 7.03 ^a^	110.2 ± 5.16 ^d^	158.7 ± 3.40 ^b^	134.1 ± 6.70 ^c^	95.5 ± 5.47 ^e^
Glycine	2.6 ± 1.43 ^d^	100.4 ± 7.96 ^b^	0.1 ± 0.01 ^d^	145.6 ± 6.98 ^a^	nd	78.7 ± 7.67 ^c^
Histidine	4.8 ± 0.55 ^d^	69.6 ± 2.13 ^b^	2.5 ± 1.14 ^d^	149.8 ± 8.00 ^a^	2.35 ± 0.15 ^d^	55.3 ± 3.32 ^c^
Isoleucine	69.5 ± 1.43 ^e^	179.0 ± 3.97 ^b^	65.7 ± 1.47 ^e^	418.9 ± 3.01 ^a^	122.2 ± 0.34 ^d^	88.0 ± 6.69 ^c^
Leucine	99.8 ± 3.00 ^e^	493.2 ± 7.48 ^b^	100.0 ± 6.51 ^e^	1330.9 ± 6.12 ^a^	166.4 ± 0.97 ^d^	434.1 ± 3.50 ^c^
Lysine	8.5 ± 1.10 ^d^	150.0 ± 7.55 ^c^	3.4 ± 0.42 ^d^	341.0 ± 4.46 ^a^	3.9 ± 0.68 ^d^	171.9 ± 8.11 ^b^
Methionine	10.0 ± 1.00 ^f^	83.3 ± 0.60 ^c^	16.8 ± 1.80 ^e^	467.6 ± 4.25 ^a^	68.8 ± 0.61 ^d^	107.6 ± 2.69 ^b^
Phenylalanine	124.0 ± 4.49 ^e^	370.6 ± 8.56 ^b^	119.5 ± 4.50 ^e^	796.1 ± 7.05 ^a^	182.5 ± 3.13 ^d^	288.3 ± 7.65 ^c^
Proline	184.0 ± 6.80 ^d^	800.5 ± 0.83 ^a^	145.4 ± 3.49 ^e^	253.8 ± 6.67 ^c^	85.8 ± 0.36 ^f^	282.4 ± 8.18 ^b^
Serine	95.2 ± 2.00 ^e^	319.0 ± 3.18 ^b^	74.8 ± 3.51 ^f^	481.8 ± 8.47 ^a^	177.5 ± 3.62 ^d^	204.5 ± 3.19 ^c^
Threonine	43.3 ± 1.00 ^e^	182.1 ± 8.70 ^c^	54.6 ± 3.51 ^e^	383.7 ± 8.81 ^a^	75.6 ± 1.18 ^d^	205.8 ± 7.40 ^b^
Tryptophan	219.1 ± 2.51 ^c^	70.5 ± 0.47 ^d^	86.3 ± 3.01 ^d^	350.0 ± 7.93 ^a^	328.0 ± 12.70 ^b^	77.9 ± 5.50 ^d^
Tyrosine	31.2 ± 1.36 ^e^	191.1 ± 6.29 ^c^	37.7 ± 1.47 ^e^	666.2 ± 6.18 ^a^	66.5 ± 1.42 ^d^	208.5 ± 5.19 ^b^
Valine	146.7 ± 2.69 ^e^	307.1 ± 90 ^b^	145.5 ± 2.31 ^e^	524.5 ± 8.60 ^a^	190.9 ± 2.77 ^d^	224.3 ± 6.05 ^c^

Note: Results are expressed as mean ± SD (*n* = 3 independent replicates) in mg/kg on a dry weight basis (DW); nd—not detected. Different superscript letters within the same row indicate statistically significant differences among samples (*p* < 0.05), according to one-way ANOVA followed by Tukey’s HSD test.

**Table 4:** Neuroactive compounds (µg/kg) of samples.

	TFP	NOFP	AFP
Histamine	2026 ± 96.21 ^a^	2055 ± 151 ^a^	1919 ± 84.95 ^a^
L-DOPA	1113 ± 75.16 ^c^	9235 ± 101 ^b^	15,272 ± 417 ^a^
Tyramine	1221 ± 7.04 ^b^	1970 ± 5.08 ^a^	1276 ± 55.94 ^b^
Serotonin	n.d	811 ± 17.04 ^a^	n.d
Kynurenine	309 ± 4.98 ^c^	531 ± 3.5 ^b^	868 ± 6.69 ^a^
Phenylethylamine	440 ± 9.1 ^a^	55 ± 11.8 ^c^	203 ± 6.57 ^b^
Tryptamine	n.d	n.d	373 ± 4.41 ^a^
Kynurenic acid	45 ± 5.21 ^c^	67 ± 7.1 ^b^	1539 ± 7.88 ^a^
Indole-3-lactic acid	n.d	1060 ± 58.34 ^b^	1372 ± 37.55 ^a^
Indole-3-acetic acid	n.d	475 ± 39.5 ^c^	n.d

Note: Values are expressed as mean ± standard deviation (*n* = 3). Different superscript letters indicate statistically significant differences within a row (*p* < 0.05). n.d—not detected, below the LOQ.

**Table 5:** Aroma compounds of fermented beverages (ng/g sample).

Aroma Compounds	TFP	NOFP	AFP
	Aldehydes		
hexanal	2405.19 ± 4.27 ^b^	2104 ± 0.66 ^c^	4377 ± 3.29 ^a^
benzaldehyde	756.85 ± 2.02 ^b^	683 ± 8.95 ^c^	1597 ± 3.14 ^a^
phenylacetaldehyde	685.25 ± 6.62 ^c^	728 ± 9.55 ^b^	1923 ± 8.36 ^a^
nonanal	280.96 ± 4.24 ^b^	278 ± 8.02 ^b^	801 ± 2.78 ^a^
E-2-octenal	126.04 ± 8.02 ^c^	149 ± 3.55 ^b^	316 ± 4.78 ^a^
E.E-2.4-decadienal	29.37 ± 5.98 ^b^	14 ± 3.35 ^c^	42 ± 2.6 ^a^
	Ketones		
2,3-butanedione (diacetyl)	502 ± 4.42 ^a^	152 ± 2.91 ^c^	241 ± 2.38 ^b^
2,3-pentanedione	35 ± 2.35 ^a^	28 ± 4.16 ^b^	26 ± 3.08 ^b^
2-heptanone	168 ± 8.50 ^b^	78 ± 3.42 ^c^	240 ± 6.22 ^a^
2-nonanone	4 ± 0.33 ^b^	3 ± 0.51 ^b^	9 ± 1.00 ^a^
	Alcohols		
1-octen-3-ol	903 ± 9.83 ^c^	1714 ± 5.08 ^a^	1538 ± 8.09 ^b^
2-phenylethanol	297 ± 6.86 ^b^	118 ± 3.34 ^c^	348 ± 6.05 ^a^
benzenemethanol	96 ± 9.75 ^c^	127 ± 6.23 ^b^	320 ± 2.04 ^a^
1-pentanol	752 ± 5.59 ^a^	386 ± 8.00 ^b^	326 ± 3.32 ^c^
	Acids		
acetic acid	1488 ± 4.93 ^c^	2201 ± 6.86 ^b^	2283 ± 7.68 ^a^
hexanoic acid	5650 ± 5.61 ^a^	3524 ± 7.27 ^c^	5456 ± 8.85 ^b^
octanoic acid	282 ± 1.15 ^b^	306 ± 3.99 ^a^	292 ± 6.6 ^b^
nonanoic acid	1957 ± 4.36 ^a^	1490 ± 4.44 ^b^	1046 ± 8.86 ^c^
	Pyrazines		
2,5-dimethylpyrazine	2 ± 0.32 ^b^	1 ± 0.05 ^b^	7 ± 0.66 ^a^
2,6-dimethylpyrazine	15 ± 0.53 ^b^	7 ± 0.55 ^c^	59 ± 3.98 ^a^
trimethylpyrazine	6 ± 0.48 ^b^	2 ± 0.26 ^c^	8 ± 0.63 ^a^
tetramethylpyrazine	5 ± 0.39 ^b^	3.2 ± 0.37 ^c^	8 ± 0.81 ^a^
	Furans		
2-methylfuran	10 ± 0.64 ^a^	4 ± 0.14 ^c^	8 ± 1.09 ^b^
2-pentylfuran	718 ± 7.22 ^b^	270 ± 5.76 ^c^	1257 ± 4.24 ^a^
2-furfural	123 ± 7.13 ^b^	120 ± 3.75 ^b^	197 ± 2.44 ^a^
	Sulfur compounds		
dimethyldisulfide	17 ± 0.62 ^a^	5 ± 0.54 ^c^	12 ± 0.65 ^b^
dimethyltrisulfide	81 ± 5.13 ^b^	65 ± 4.06 ^c^	120 ± 1.92 ^a^
methional	31 ± 0.85 ^b^	38 ± 4.09 ^b^	201 ± 6.08 ^a^
	Lactones		
gamma-hexalactone	244 ± 5.20 ^a^	157 ± 1.81 ^c^	224 ± 5.99 ^b^
gamma-octalactone	34 ± 1.64 ^b^	50 ± 8.74 ^b^	71 ± 8.56 ^a^
gamma-nonalactone	101 ± 7.13 ^c^	127 ± 2.76 ^b^	172 ± 5.38 ^a^

Note: Values are expressed as mean ± standard deviation (*n* = 3). Different superscript letters indicate statistically significant differences within the row (*p* < 0.05).

## Data Availability

The original contributions presented in this study are included in the article/Supplementary Materials. Further inquiries can be directed to the corresponding author.

## Supplementary Materials

The following supporting information can be downloaded at: https://www.mdpi.com/article/10.3390/foods15173146/s1.

## Abbreviations

The following abbreviations are used in this manuscript:

TG	Triticale grain
NOG	naked oat grain
AG	amaranth grain
GABA	γ-aminobutyric acid
CNS	central nervous system
TFP	triticale fermented product
NOFP	naked oat fermented product
AFP	amaranth fermented product

## References

[1] Cazcarro I., López-Morales C.A., Duchin F. (2019). The Global Economic Costs of Substituting Dietary Protein from Fish with Meat, Grains and Legumes, and Dairy. J. Ind. Ecol..

[2] Wang X., Wang L., Wei X., Xu C., Cavender G., Lin W., Sun S. (2025). Invited Review: Advances in Yogurt Development—Microbiological Safety, Quality, Functionality, Sensory Evaluation, and Consumer Perceptions across Different Dairy and Plant-Based Alternative Sources. J. Dairy. Sci..

[3] Shah N.P. (2017). Yogurt in Health and Disease Prevention.

[4] Nionelli L., Coda R., Curiel J.A., Poutanen K., Gobbetti M., Rizzello C.G. (2014). Manufacture and Characterization of a Yogurt-like Beverage Made with Oat Flakes Fermented by Selected Lactic Acid Bacteria. Int. J. Food Microbiol..

[5] Whitehead A., Beck E.J., Tosh S., Wolever T.M. (2014). Cholesterol-Lowering Effects of Oat *β*-Glucan: A Meta-Analysis of Randomized Controlled Trials. Am. J. Clin. Nutr..

[6] Demir H., Yıldırım G., Simsek M. (2021). Effect of Oat Milk Pasteurization Type on the Characteristics of Yogurt. LWT-Food Sci. Technol..

[7] Marsh A.J., Hill C., Ross R.P., Cotter P.D. (2014). Fermented Beverages with Health-Promoting Potential: Past and Future Perspectives. Trends Food Sci. Technol..

[8] Schmidt D., Verruma-Bernardi M.R., Forti V.A., Borges M.T.M.R. (2023). Quinoa and Amaranth as Functional Foods: A Review. Food Rev. Int..

[9] Matejčeková Z., Liptáková D., Valík Ľ. (2015). Fermentation of Milk- and Water-Based Amaranth Mashes. Acta Chim. Slovaca.

[10] Shevkani K., Singh N., Rana J.C., Kaur A. (2014). Relationship between Physicochemical and Functional Properties of Amaranth (Amaranthus Hypochondriacus) Protein Isolates. Int. J. Food Sci. Technol..

[11] Hamid, Kathuria D., Gautam S., Suri S., Jaiswal A.K., Singh J., Kaur S., Rasane P., Singh J. (2024). Triticale. Cereals and Nutraceuticals.

[12] Arya P., Vaidya D., Devi S., Kaushal M., Myathtwe H., Devi D., Gupta A. (2025). Fermentation-Driven Bioactive Enhancement in Cereal Grains: Mechanisms, Nutritional Improvements, and Functional Food Applications. Trends Food Sci. Technol..

[13] Krebs G., Müller M., Becker T., Gastl M. (2019). Characterization of the Macromolecular and Sensory Profile of Non-Alcoholic Beers Produced with Various Methods. Food Res. Int..

[14] Marco M.L., Heeney D., Binda S., Cifelli C.J., Cotter P.D., Foligné B., Gänzle M., Kort R., Pasin G., Pihlanto A. (2017). Health Benefits of Fermented Foods: Microbiota and Beyond. Curr. Opin. Biotechnol..

[15] Adebo J.A., Njobeh P.B., Gbashi S., Oyedeji A.B., Ogundele O.M., Oyeyinka S.A., Adebo O.A. (2022). Fermentation of Cereals and Legumes: Impact on Nutritional Constituents and Nutrient Bioavailability. Fermentation.

[16] Richard D.M., Dawes M.A., Mathias C.W., Acheson A., Hill-Kapturczak N., Dougherty D.M. (2009). L-Tryptophan: Basic Metabolic Functions, Behavioral Research and Therapeutic Indications. Int. J. Tryptophan Res..

[17] Zhang Q., Zhu L., Li H., Chen Q., Li N., Li J., Zhao Z., Xiao D., Tang T., Bi C. (2024). Insights and Progress on the Biosynthesis, Metabolism, and Physiological Functions of Gamma-Aminobutyric Acid (GABA): A Review. PeerJ.

[18] Fernstrom J.D., Fernstrom M.H. (2007). Tyrosine, Phenylalanine, and Catecholamine Synthesis and Function in the Brain. J. Nutr..

[19] Saha Turna N., Chung R., McIntyre L. (2024). A Review of Biogenic Amines in Fermented Foods: Occurrence and Health Effects. Heliyon.

[20] Maintz L., Novak N. (2007). Histamine and Histamine Intolerance. Am. J. Clin. Nutr..

[21] Schmidt R.W., Thompson M.L. (2016). Glycinergic Signaling in the Human Nervous System: An Overview of Therapeutic Drug Targets and Clinical Effects. Ment. Health Clin..

[22] Wang Y., Wu J., Lv M., Shao Z., Hungwe M., Wang J., Bai X., Xie J., Wang Y., Geng W. (2021). Metabolism Characteristics of Lactic Acid Bacteria and the Expanding Applications in Food Industry. Front. Bioeng. Biotechnol..

[23] Thage B.V., Broe M.L., Petersen M.H., Petersen M.A., Bennedsen M., Ardö Y. (2005). Aroma Development in Semi-Hard Reduced-Fat Cheese Inoculated with *Lactobacillus paracasei* Strains with Different Aminotransferase Profiles. Int. Dairy. J..

[24] Bancalari E., Savo Sardaro M.L., Levante A., Marseglia A., Caligiani A., Lazzi C., Neviani E., Gatti M. (2017). An Integrated Strategy to Discover *Lactobacillus casei* Group Strains for Their Potential Use as Aromatic Starters. Food Res. Int..

[25] Xu L., Mei X., Wu G., Karrar E., Jin Q., Wang X. (2022). Inhibitory Effect of Antioxidants on Key Off-Odors in French Fries and Oils and Prolong the Optimum Frying Stage. LWT.

[26] Beltrán-Barrientos L.M., Garcia H.S., Reyes-Díaz R., Estrada-Montoya M.C., Torres-Llanez M.J., Hernández-Mendoza A., González-Córdova A.F., Vallejo-Cordoba B. (2019). Cooperation between Lactococcus Lactis NRRL B-50571 and NRRL B-50572 for Aroma Formation in Fermented Milk. Foods.

[27] Mæhre H.K., Dalheim L., Edvinsen G.K., Elvevoll E.O., Jensen I.-J. (2018). Protein Determination—Method Matters. Foods.

[28] Amare E., Mouquet-Rivier C., Rochette I., Adish A., Haki G.D. (2016). Effect of Popping and Fermentation on Proximate Composition, Minerals and Absorption Inhibitors, and Mineral Bioavailability of Amaranthus Caudatus Grain Cultivated in Ethiopia. J. Food Sci. Technol..

[29] Thiex N., Novotny L., Crawford A. (2012). Determination of Ash in Animal Feed: AOAC Official Method 942.05 Revisited. J. AOAC Int..

[30] DuBois M., Gilles K.A., Hamilton J.K., Rebers P.A., Smith F. (1956). Colorimetric Method for Determination of Sugars and Related Substances. Anal. Chem..

[31] Lee S.C., Prosky L., De Vries J.W. (1992). Determination of Total, Soluble, and Insoluble Dietary Fiber in Foods—Enzymatic-Gravimetric Method, MES-TRIS Buffer: Collaborative Study. J. AOAC Int..

[32] Huang D., Ou B., Prior R.L. (2005). The Chemistry behind Antioxidant Capacity Assays. J. Agric. Food Chem..

[33] Re R., Pellegrini N., Proteggente A., Pannala A., Yang M., Rice-Evans C. (1999). Antioxidant Activity Applying an Improved ABTS Radical Cation Decolorization Assay. Free Radic. Biol. Med..

[34] Kubola J., Siriamornpun S., Meeso N. (2011). Phytochemicals, Vitamin C and Sugar Content of Thai Wild Fruits. Food Chem..

[35] Yılmaz C., Ozdemir F., Gökmen V. (2019). Investigation of Free Amino Acids, Bioactive and Neuroactive Compounds in Different Types of Tea and Effect of Black Tea Processing. LWT.

[36] Yılmaz C., Ecem Berk Ş., Gökmen V. (2024). Effect of Different Stress Conditions on the Formation of Amino Acid Derivatives by Brewer’s and Baker’s Yeast during Fermentation. Food Chem..

[37] Kocadağlı T., Göncüoğlu Taş N., Hamzalıoğlu A., Gökmen V. (2025). Formation of Acrylamide and Aroma Compounds in Biscuits and Crackers Prepared with Sprouted Wheat Wholemeal. Food Chem..

[38] Moore J.F., DuVivier R., Johanningsmeier S.D. (2021). Formation of γ-Aminobutyric Acid (GABA) during the Natural Lactic Acid Fermentation of Cucumber. J. Food Compos. Anal..

[39] Cáceres P.J., Peñas E., Martínez-Villaluenga C., García-Mora P., Frías J. (2019). Development of a Multifunctional Yogurt-like Product from Germinated Brown Rice. LWT.

[40] Manassero C.A., Añón M.C., Speroni F. (2020). Development of a High Protein Beverage Based on Amaranth. Plant Foods Hum. Nutr..

[41] Aydar E.F., Tutuncu S., Ozcelik B. (2020). Plant-Based Milk Substitutes: Bioactive Compounds, Conventional and Novel Processes, Bioavailability Studies, and Health Effects. J. Funct. Foods.

[42] Arise A.K., Alashi A.M., Nwachukwu I.D., Ijabadeniyi O.A., Aluko R.E., Amonsou E.O. (2016). Antioxidant Activities of Bambara Groundnut (*Vigna subterranea*) Protein Hydrolysates and Their Membrane Ultrafiltration Fractions. Food Funct..

[43] Lim E.-S. (2025). Antioxidant Capacity of Small Black Soymilk Fermented with ROS-Resistant Probiotics. Food Sci. Biotechnol..

[44] Dong B., Yin X., Shan X., Su J., Wei Z., Yao Y., Zhao G. (2026). Indole Derivatives from Lactic Acid Bacteria in Fermented Foods: Biosynthesis, Functional Activities, and Implications for Food Quality and Human Health. Trends Food Sci. Technol..

[45] Ling L., Pang M., Luo H., Cheng W., Jiang K., Wang Y. (2023). Antifungal Activity of Diacetyl, a Volatile Organic Compound, on *Trichoderma lixii* F2 Isolated from Postharvest Lanzhou Lily Bulbs. Food Biosci..

[46] Gobbetti M., De Angelis M., Di Cagno R., Polo A., Rizzello C.G. (2020). The Sourdough Fermentation Is the Powerful Process to Exploit the Potential of Legumes, Pseudo-Cereals and Milling by-Products in Baking Industry. Crit. Rev. Food Sci. Nutr..

[47] De Angelis M., Rizzello C.G., Alfonsi G., Arnault P., Cappelle S., Di Cagno R., Gobbetti M., Tossut P. (2007). Use of Sourdough Lactobacilli and Oat Fibre to Decrease the Glycaemic Index of White Wheat Bread. Br. J. Nutr..

[48] Gao K., Mu C.-L., Farzi A., Zhu W.-Y. (2020). Tryptophan Metabolism: A Link Between the Gut Microbiota and Brain. Adv. Nutr..

[49] Hou Y., Li J., Ying S. (2023). Tryptophan Metabolism and Gut Microbiota: A Novel Regulatory Axis Integrating the Microbiome, Immunity, and Cancer. Metabolites.

[50] Shewry P.R., Halford N.G. (2002). Cereal Seed Storage Proteins: Structures, Properties and Role in Grain Utilization. J. Exp. Bot..

[51] Nanson N.J., Fields M.L. (1984). Influence of Temperature of Fermentation on the Nutritive Value of Lactic Acid Fermented Cornmeal. J. Food Sci..

[52] Chen Y.-M., Lin C.-L., Wei L., Hsu Y.-J., Chen K.-N., Huang C.-C., Kao C.-H. (2016). Sake Protein Supplementation Affects Exercise Performance and Biochemical Profiles in Power-Exercise-Trained Mice. Nutrients.

[53] Yabuki Y., Ohashi M., Imagawa F., Ishiyama K., Beier M.P., Konishi N., Umetsu-Ohashi T., Hayakawa T., Yamaya T., Kojima S. (2017). A Temporal and Spatial Contribution of Asparaginase to Asparagine Catabolism during Development of Rice Grains. Rice.

[54] Barba de la Rosa A.P., Barba Montoya A., Martínez-Cuevas P., Hernández-Ledesma B., León-Galván M.F., De León-Rodríguez A., González C. (2010). Tryptic Amaranth Glutelin Digests Induce Endothelial Nitric Oxide Production through Inhibition of ACE: Antihypertensive Role of Amaranth Peptides. Nitric Oxide.

[55] Engels W., Siu J., van Schalkwijk S., Wesselink W., Jacobs S., Bachmann H. (2022). Metabolic Conversions by Lactic Acid Bacteria during Plant Protein Fermentations. Foods.

[56] Liu S.-Q., Holland R., Crow V.L. (2003). The Potential of Dairy Lactic Acid Bacteria to Metabolise Amino Acids via Non-Transaminating Reactions and Endogenous Transamination. Int. J. Food Microbiol..

[57] Ibieta G., Ortiz-Sempértegui J., Grey C., Peñarrieta J.M., Linares-Pastén J.A. (2025). γ-Aminobutyric Acid (GABA) Production and Soluble Free Amino Acid Profile Change in Andean Seeds by Levilactobacillus Brevis Fermentation. Front. Nutr..

[58] Gainetdinov R.R., Hoener M.C., Berry M.D. (2018). Trace Amines and Their Receptors. Pharmacol. Rev..

[59] Cheng L., Liu J., Chen Z. (2021). The Histaminergic System in Neuropsychiatric Disorders. Biomolecules.

[60] De Deurwaerdère P., Di Giovanni G., Millan M.J. (2017). Expanding the Repertoire of L-DOPA’s Actions: A Comprehensive Review of Its Functional Neurochemistry. Prog. Neurobiol..

[61] Berry M.D. (2004). Mammalian Central Nervous System Trace Amines. Pharmacologic Amphetamines, Physiologic Neuromodulators. J. Neurochem..

[62] Ede-Çïntesun E., Yaman M. (2026). Targeted Profiling of Tryptophan- and Tyrosine-Derived Metabolites in Traditionally Fermented Foods. Plant Foods Hum. Nutr..

[63] Baraniak J., Kania-Dobrowolska M. (2022). The Dual Nature of Amaranth—Functional Food and Potential Medicine. Foods.

[64] Tapia S.M., Moreno-Ruiz A., Tres A., Moreno-Yerro N., Arrubla P., Gastón-Lorente M., Mohedano M.L., Virto R., Fratebianchi D. (2025). Butter Aroma Compounds in Plant-Based Milk Alternatives through Fermentation: Screening of Potential Starter Cultures and Enhanced Production in Oat Milk with *Lactococcus cremoris*. LWT.

[65] Hosken B.d.O., Melo Pereira G.V., Lima T.T.M., Ribeiro J.B., de Magalhães Júnior W.C.P., Martin J.G.P. (2023). Underexplored Potential of Lactic Acid Bacteria Associated with Artisanal Cheese Making in Brazil: Challenges and Opportunities. Fermentation.

[66] Lee S.M., Oh J., Hurh B.-S., Jeong G.-H., Shin Y.-K., Kim Y.-S. (2016). Volatile Compounds Produced by Lactobacillus paracasei During Oat Fermentation. J. Food Sci..

[67] He Z., Zhang H., Wang T., Wang R., Luo X. (2022). Effects of Five Different Lactic Acid Bacteria on Bioactive Components and Volatile Compounds of Oat. Foods.

