# Supplementary material for: Study of the Formation of Neuromodulatory Compounds in Fermented Products Based on Triticale, Naked Oat, and Amaranth Grains
Source: Foods. 2026 Sep 4;15(17):3146. doi: 10.3390/foods15173146 (PMC13564608; doi:10.3390/foods15173146)
Supplement: Supplementary file 1 [file foods-15-03146-s001.zip › foods-4512376-supplementary.pdf]

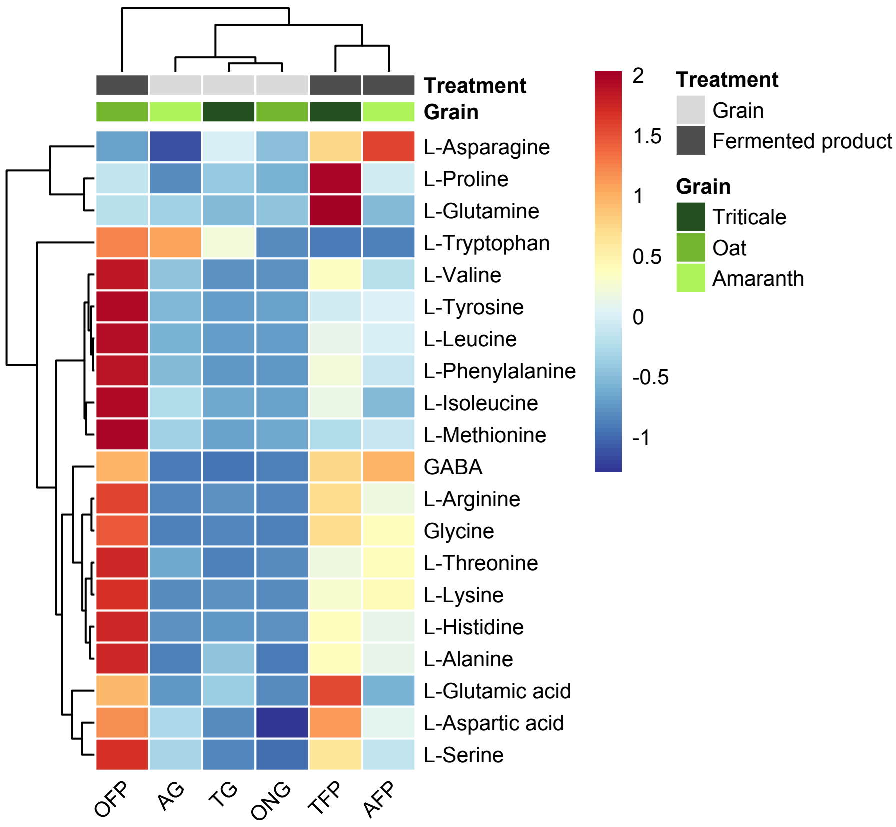

Treatment
Grain
L-Asparagine
L-Proline
L-Glutamine
L-Tryptophan
L-Valine
L-Tyrosine
L-Leucine
L-Phenylalanine
L-Isoleucine
L-Methionine
GABA
L-Arginine
Glycine
L-Threonine
L-Lysine
L-Histidine
L-Alanine
L-Glutamic acid
L-Aspartic acid
L-Serine
OFP
AG
TG
ONG
TFP
AFP
2
1.5
1
0.5
0
-0.5
-1
Treatment
Grain
Fermented product
Grain
Triticale
Oat
Amaranth
